# Global trade of South Korea in competitive products and their impact on regional dependence

**DOI:** 10.1371/journal.pone.0267695

**Published:** 2022-05-04

**Authors:** Dongsuk Kang, Pil-sun Heo, Duk Hee Lee

**Affiliations:** 1 Department of Business Management, College of Social Science, Gangneung-Wonju National University (GWNU), Gangneung, Republic of Korea; 2 Economics of Technology Research Division, Future Technology & Strategy Research Laboratory, Electronics and Telecommunications Research Institute (ETRI), Daejeon, Republic of Korea; 3 School of Business and Technology Management, College of Business, Korea Advanced Institute of Science and Technology (KAIST), Daejeon, Republic of Korea; Tohoku University, JAPAN

## Abstract

The economic growth of a nation under the competition among countries can result from the interaction of the diversity and complexity of product export and import relations on the globe. This research aims to evaluate the competitiveness of South Korea’s trading products and its partner countries’ dependency by implementing a product and partner-based analysis. This research raises questions about the transactional positions of products and trading partners based on the diversification of import-export relations of South Korea. This study utilizes the matrix of products and trading partners from the Korean product export and import data from 1995 to 2015. The research analyzes Korea’s product competitiveness and dependency of trading countries on Korea using the Revealed Comparative Advantage (RCA) and a nonlinear iterative method (NIM). The study finds that the products of several manufacturing industries showed a large production scale. From the global perspective, the trade dependency on Korea was high in Asia and in Africa and South America where the portion of underdeveloped or developing countries is relatively large. This research suggests that Korea may face difficulties of continuing growth if it maintains or intensifies its trade relation pattern under the environment of rapidly changing technology and economy. Therefore, diversification and mutual complementarity could be important for the export of promising products and industrial development policy.

## Introduction

The global contagion of COVID-19 (or SARS-CoV-2), which began at the end of 2019, its economic shocks, concerns about the disruption of world trade, and human mobility could explain the current situation in which many nations interact (in)directly with each other from the viewpoint of global value chains [1]. The consequent blockade or standstill of certain regions and countries such as Italy [2] for the prevention and control of infection of COVID-19 would denote the impact of global interdependence and trade portfolio resulting from the densely connected relationship among many countries.

This research aims to evaluate the competitiveness of South Korea’s trading products and its partner countries’ dependency by implementing a product and partner-based analysis. This study analyzes the transactional positions of products-trading partners’ (e.g., countries and territories. hereinafter partners) export structure focusing on the diversification of import-export relations of the Republic of Korea (Hereinafter “Korea”). This research empirically investigates the case study of Korea as it has several meaningful points of research values, such as the exemplary analysis of the economic complexity of the Netherlands [3].

Above all, Korea ranked high in terms of economic capabilities and global trade, for example, twelfth in GDP (US$ 1.62 trillion in 2018), tenth in product imports (US$ 509 billion in 2018), and fifth in product exports (US$ 617 billion in 2018) around the world [4]. Korea encountered the Asian financial crisis in 1997, and it overcame several economic shocks (e.g., the Asian crisis and the United States of America financial crisis in 2007) with comparative ease [5]. It has also been recognized as one of the leading countries in the field of electronic government (e-government), the ICT industrial infrastructure and mobile businesses [6, 7]. Changing the Korean trade relationship would be a topic of geopolitical research that can affect trade and foreign policy related to several major countries (e.g., the USA, China, and Japan).

Therefore, this research establishes the following two research questions concerning the individual product competitiveness of the nation and the dependency of major trading partners: **Research question 1 (i.e., RQ 1)**. Which are the important products in Korea’s trade relationship, and how can they be measured? **RQ 2**. Which are the major trading partners in the transactional relationship, and how can they be measured? Answers to these questions could be helpful in establishing the portfolio strategy and such status in the global market for the products as basis for sustained growth and development under the changing environment of trade competition.

This research utilized the trade data of transactional partners during the period 1995–2015 for Korea, which has an export-led economic system, using the metrics on the product competitiveness and dependency of the trading partner country based on the nonlinear iterative method (hereinafter **NIM**) derived from the product-country matrix. This research revised the methodology of Tacchella, Cristelli [8]’s economic complexity and offered new measurement of product fitness (competitiveness) and its trading partners’ dependency in a nation’s viewpoint (Please see the **Literature Review**). The result showed a large production scale in several manufacturing industries. In general, Korea has maintained significant trade relations with Asian countries that have similar physical space and socioeconomic environment and countries in Africa and South America where the portion of underdeveloped countries is relatively large.

As a contribution, this research identifies the import and export structure of products at the national level and assesses the fitness of the export products and the dependency of the trading partner country vis-à-vis Korea. This finding can highlight that a country’s diversification with its export products is an important survival strategy and a source of competitiveness in the global competitive environment. Therefore, the diversification of export products and trading countries can be one of the sustainable economic growth strategies under the dynamically evolving global economic system built on science and engineering-based innovation (e.g., the Fourth Industrial Revolution [4IR] and the development of Artificial Intelligence [AI]).

### Literature review on a nation’s economic growth and its competitiveness in trade diversification

Managing dynamic portfolio of product exports of a nation could be a critical factor in its economic development. Earlier studies in the field of global trades explain the increase in wealth of a nation as a consequence of an agent’s (e.g., a country) economic effect on its relative advantage to others and its division of work and specialization [8, 9]. From this perspective (e.g., Adam Smith and Ricardian approach), many countries would export and import very differentiated products from each other. However, recent empirical analyses on the trade networks of a nation have found conflicting results that developed countries export various products with different qualities or complexities, and developing countries export a small number of products that other countries manufacture [8–11].

The new methodological stream of international trade and economic growth is the analysis of the economic complexity of countries with a trade network that utilizes the transactional matrix of nations and their exporting products [8, 9]. This methodology was first named the Method of Reflections in research conducted by Hidalgo and Hausmann [9] (hereinafter MR). Subsequent research has developed it into a methodology family called economic complexity [8, 12]. This methodology would be fundamentally different from the existing econometric studies that capture the relationship between economic performance of a nation and its inputs (e.g., labor, capital, technological capabilities) with their aggregate equations [9, 11]. The new methodology analyzes the relationship as the international trade web that nations utilize their non-tradable capabilities and resources and export their competitive products from the perspective of the complex networks of countries, such as bipartite or tripartite network of countries (their hidden production competences), and products [9]. The methodologies of MR and economic complexity commonly investigate the competitive advantage of a nation *c*’s product *p* under its superior condition (i.e., *RCA*_*pc*_ ≥ 1) of Revealed Comparative Advantage (RCA) [8–11]. MR also could be similar to the measurement of Google’s Page Rank capturing the relative important position in the network data, which seems to be a kind of linear analytic tool [13].

The economic complexity methodology analyzes the competitive values of a nation and its export product(s) based on information on the number of export goods of the nation (i.e., the diversification of a nation) and the number of other countries that export the same goods (i.e., the ubiquity of a product) [9]. Empirically, nations with various export products are less likely to manufacture goods produced and exported by other nations [9]. Conversely, countries with few export products have relatively low competitive goods capabilities from viewpoint of RCA; the countries consequently could encounter the quiescence trap [11] which has low incentives to develop capabilities for producing and exporting various competent products.

Research on economic complexity can be categorized into two groups: Hidalgo and Hausmann [9]’s linear analysis (MR) and Tacchella, Cristelli [8]’s nonlinear analysis (hereinafter TC). MR can reflect qualitative information about exporting products (e.g., a product’s complexity and the competitiveness of its exporter) and overcome the shallow analysis of the existing diversification measurement (e.g., Herfindahl–Hirschman Index [HHI], Entropy Index) [11]. For example, these indices hardly identify the information difference between the export backet A (apples: semi-conductors = 9:1) and basket B (apples: semi-conductors = 1:9) [11]. However, several studies have repeatedly criticized MR for inaccurately measuring the competitiveness of the exporting product by averaging the competence of its exporting country on the basis of its linear calculation [3, 8, 10]. As an alternative to MR, Tacchella, Cristelli [8]’s nonlinear analysis can gauge the competitiveness or fitness of the export product by taking the reciprocal value of the competence of the exporter because the competence of the exporter can be measured as the sum of competitiveness of its products.

Recent research has investigated viewpoints of competitive exporting products and its trading partners at the national/regional level with the Method of Reflection (MR) and nonlinear analysis [14–16]. At the regional level, identifying and investigating the core products and key partners could be useful for industrial restructuring of development plans and regional innovation. For example, Canada’s key exporting products were natural resources based on goods (e.g., wood pulp, oats, rapeseed) with high RCA but low product complexity calculated by MR with its major traders in 2017 being the United States (75.9%), China (18.2%), and the United Kingdom (13.6%) [14]. This finding can suggest a nation/region (e.g., Canada, Quebec) needs to evolve its competitive edge of products and partners toward a kind of diversified portfolio of exports with high complexity and various trading counterparties [14].

The matrix of exporting products and transactional regions at one nation’s level could also offer insights in capturing leading regions and relieving provincial discrepancy by trades [15, 16]. Several dominant tendencies about high economic complexities of regional transactions were in the costal and capital city-neighboring provinces [15, 16]. This high level of industrial economic complexity and the regional fitness could be the outcomes of increasing urbanization and trade in China [15]. Further, the economic complexity by MR could also contribute to the regional growth by GDP in Mexico [16].

The aforementioned literature has mainly investigated the dynamic relationship between exporting countries and trading products [8–12, 17, 18]. Several studies offer findings of exporting regions and goods, but their findings appear to highlight the features of the trading regions [3, 15, 16]. In this research, we newly analyze the relationship of exporting products and their major partners (importing countries) at a nation’s viewpoint with the measures of product fitness and country/region’s dependence. The “product fitness (in a nation)” in this study calculates the number of products that the nation exports to other countries with RCA in the matrix of its products and importers in a nation. This measurement could be similar to the measurement of a nation’s fitness with RCA where it exports competitive products to other countries in the matrix of products as well as globally in other study of economic complexity index [3, 8, 10, 12, 17, 19]. This variation of fitness is applicable to a region’s fitness in the trade matrix between provinces in a nation [15].

This study also suggests the measurement of “dependency of countries/regions in an exporting nation”, which is the number of competitive products imported because of no local domestic production or just imported from other countries. This measurement could be an analogy to the calculation of product complexity in the product matrix, which implies the product’s low level of complexity produced by countries in other studies of economic complexity index [3, 8, 10, 12, 17, 19].

In summary, this research analyzes the competitiveness and competence of exporting products of Korea by revising Tacchella, Cristelli [8]’s nonlinear methodology of economic complexity. The existing literature on economic complexity has focused on the analysis of multinational competitiveness [8–11]. These analyses can easily capture the comparative status of the competence of nations, but the analyses can rarely investigate in-depth findings on the relative comparison between products and key partners across the nation. Furthermore, finding out about the dynamics of competitive goods and their importers over time could be important for a country that may have the opportunity to promote its strategic industries and negotiate with others, such as making concessions of exports and imports in concluding its Free Trade Area (FTA).

## Methodology and data

### Overview

**Fig 1:**
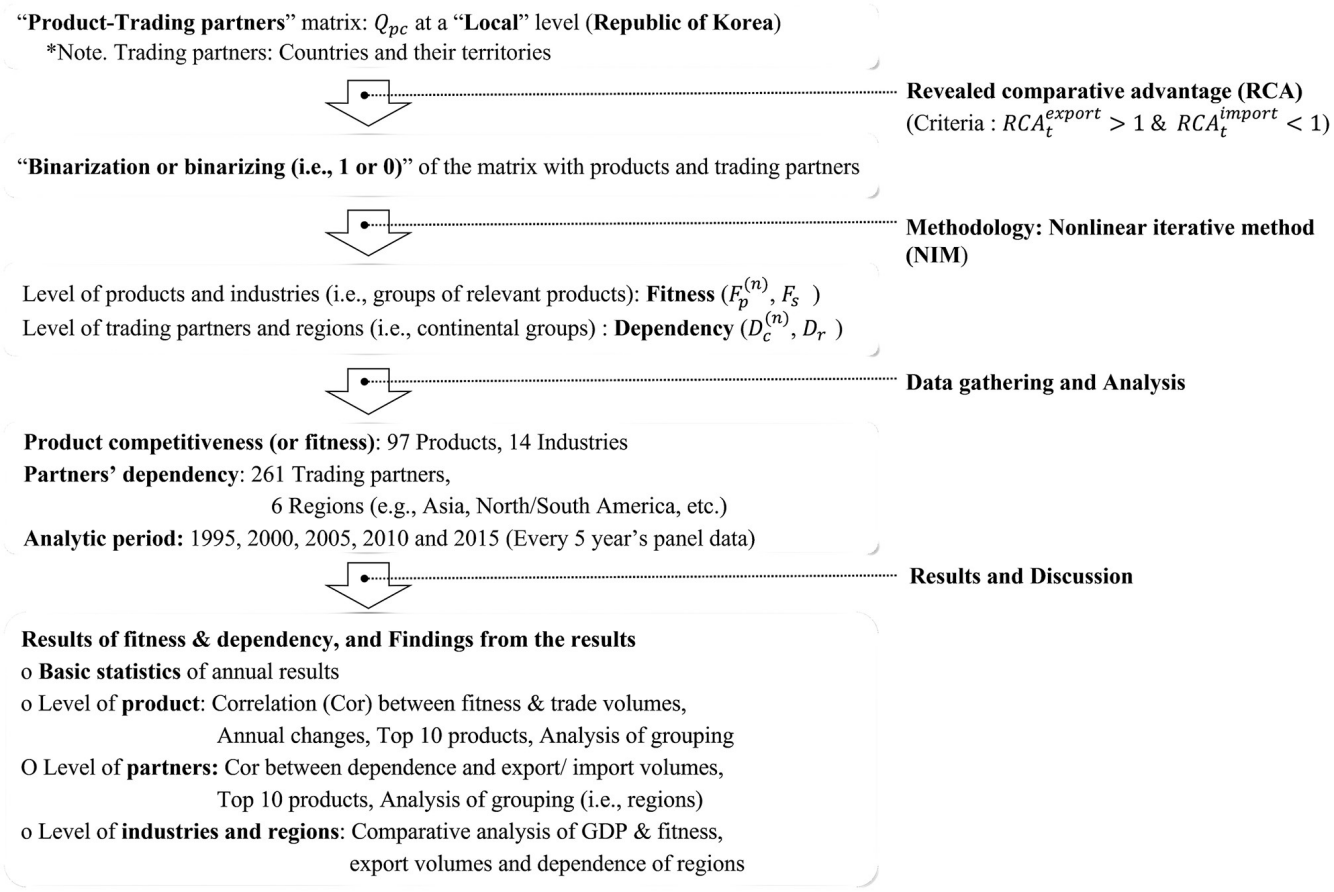
The methodological framework and analytic flow of this research.

This study utilizes international trade data between Korea and its trading partners over an extended period of time and applied various complementary methodologies to analyze the competitiveness of Korean products and the dependence of trading countries on Korea (Fig 1). This research defines trading partners as transactional countries and their territories because this definition of the partners could accurately reflect their physical distances or geological boundaries (e.g., the identical, different, or distant continent) from Korea than the sole definition of trading countries.

### Data matrix of product and trading partners with Revealed Comparative Advantage (RCA)

**Fig 2:**
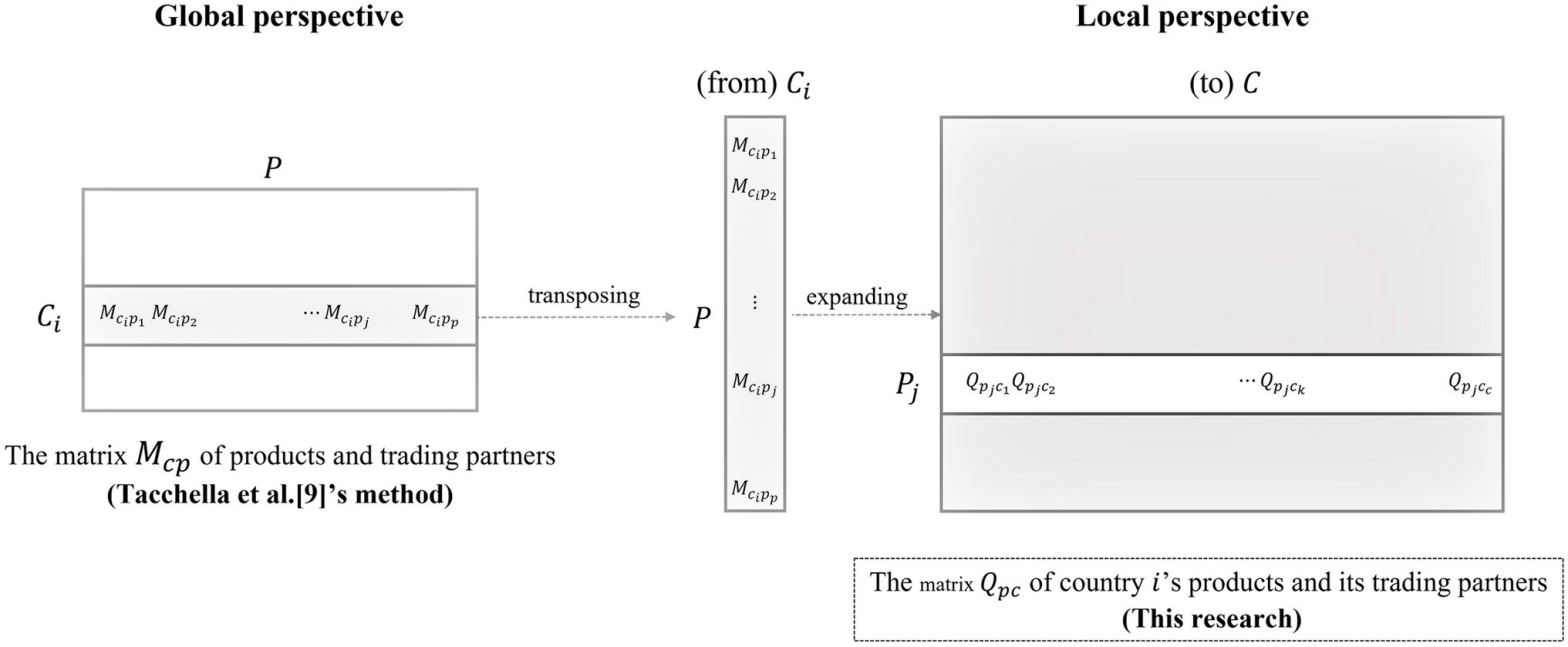
Relationship between global country-product matrix *M*_*cp*_ and local product-country matrix *Q*_*pc*_.

Recent streams of several representative research have analyzed the export data for each product in a trading partner [3, 8, 9, 17, 18]. Such analysis could be expandable to a matrix consisting of the import and export data at a local level of a specific nation. This matrix of imported (or exported) products and trading partners could measure the product’s competitiveness at the national level and dependency of the trading partners at the viewpoint of a country. The country in matrix *M* refers to any of the countries exporting the product, whereas the country in matrix *Q* refers to any trading countries which imports or exports the product from/to. The *i*^th^ row of a trading partner and products’ matrix *M*(*c* × *p*), which represents the export (or import) data of a product, shows the export (or import) volume of the product in a trading partner *i*, and the export (or import) data of each product of this partner can be expanded to the product-trading partner matrix *Q*(*p* × *c*) (Fig 2). Since this study considers full dataset of import and export information, *Q* encompasses both *Q*^*export*^ and *Q*^*import*^. The *j*^*th*^ row of matrix *Q* contains the export (import) data of product *j* with each trading partner (Eqs [hereinafter Eqs] 1–3).

$$C_{i}^{e}=\sum_{c=c_{i},p}M_{cp}=\sum_{p,c}Q_{pc}$$ (1)

$$C_{ij}^{e}=M_{c_{i}p_{j}}=\sum_{p=p_{j},c}Q_{pc}$$ (2)

$$C_{ijk}^{e}=Q_{p_{j}c_{k}}$$ (3)

※ Notes. $C_{i}^{e}$: a trading partner *i*’s total exports. $C_{ij}^{e}$: total exports of a trading partner *i*’s product *j*. $C_{ijk}^{e}$: exports of a trading partner *i*’s product *j* to the transactional counterparty *k*.

This research binarizes the semi-positive matrix of *Q*, which shows the network structure of the product-trading partners, using the Revealed Comparative Advantage (RCA) [20]. This method applies RCA formula (to each export (*Q*^*export*^) and import (*Q*^*import*^) data element (Eq 4). In particular, $Q_{p_{j}c_{k}}$ equals to one if country *i* has a RCA of its exporting product *j* (i.e., $RCA_{p_{j}c_{k}}^{export}\geq 1$) and if the country has also a RCA of its importing product *j* (i.e., $RCA_{p_{j}c_{k}}^{import}\leq 1$). If the country does not have any RCA in the aspect of exporting or importing the product, $Q_{p_{j}c_{k}}$ equals to zero in the matrix of products and trading partners.

This research newly defines $Q_{p_{j}c_{k}t}=1$ that a product *p*_*j*_ in which Korea exports to other partners that have the dual competitive advantage of exporting and importing to and from its trading partners *c*_*k*_ at *t*. This study also adopts the import condition of RCA (i.e., $RCA_{p_{j}c_{k}t}^{import}\leq 1$), which could reflect the volume of the importing products. These two conditions could minimize or control the possibility of a country’s intermediate (or brokerage) trade, which could exaggerate the meaning of RCA in exporting products. Although this method of RCA (Eq 4) could favor some countries with more absolute trade volumes of exporting natural resources (e.g., crude oil) than diversified exporters with various products [12], the method would be reasonable for Korea and other trading partners lacking of “resource curse (or paradox of plenty resources)” to investigate their relative competitiveness of exporting products.

$$RCA_{p_{j}c_{k}t}^{d}=\left(Q_{p_{j}c_{k}t}^{d}/Q_{p_{j}.t}^{d}\right)/\left(Q_{.c_{k}t}^{d}/Q_{‥t}^{d}\right)$$ (4)

※ Notes. $RCA_{p_{j}c_{k}t}^{d}$: The RCA of a trading partner *i*’s product *j* to its counterparty *k* at *t* in the deal of export or import (i.e., *d* means the trade of export or import).

$Q_{p_{j}c_{k}t}^{d}$: The trade amount of a partner *i*’s product *j* to its counterparty *k* at *t* in the aspect of export (or import).
$Q_{p_{j}.t}^{d}$: The trade amount of a partner *i*’s product *j* to all counterparties at *t* in the aspect of export (or import).
$Q_{.c_{k}t}^{d}$: The trade amount of a partner *i*’s all products to its counterparty *k* at *t* in the aspect of export (or import).
$Q_{‥t}^{d}$: The trade amount of all products all over the world at *t* in the aspect of export (or import).

### The measurement of a product competitiveness and a trading partners’ dependency

This research analyzes the competitiveness of a trade partner’s exporting products and dependency of its counterparties by measuring the imported and exported products from the country’s viewpoint. This study revises Tacchella, Cristelli [8] and Cristelli, Gabrielli [12]’s methodology about assessing national fitness and product’s complexity in order to evaluate the competitiveness of Korea’s products and its trading partners’ dependency. This methodology would be a kind of nonlinear estimation of variables as the slope or coefficient of each variable depends on the other variable (Eqs 7 and 8).

In the matrix *Q*_*pc*_ of products and trading partners (Fig 2 and Eqs 1–3), the diversification of products produced by a country is the number (*h*_*p*_ in Eq 5) of imported or exported to/ from trading partners where the product is competitive and the numerator (Eq 7) of a product’s fitness. The number of competitive products in the trading partner (connectivity) is the zero-order estimate (*h*_*c*_ in Eq 6) of dependency indicating how the trading partner depends on the analyzed country. That is, *h*_*p*_ and *h*_*c*_ represent the degree of node *p* and *c* in a bipartite network of products (*p*) and trading partners (*c*), and these metrics indicate the diversification of a product and the connectivity of a trading partner [12].


$$h_{p}=\sum_{c}Q_{pc}$$ (5)

$$h_{c}=\sum_{p}Q_{pc}$$ (6)

This research originally suggests the matrix *Q*_*pc*_ of local products and countries and defines two metrics of product fitness and a trading partner’s dependency in the aspect of Korea, which is different from the previous research [8, 10, 12] suggesting the measurements of transactional countries’ fitness and exporting product’s complexity. With the condition of the binarization (i.e., $Q_{p_{j}c_{k}t}=1$ if the relevant conditions of $RCA_{p_{j}c_{k}t}^{export}\geq 1$ and $RCA_{p_{j}c_{k}t}^{import}\leq 1$ fulfill. *t* = the year of 1995, 2000, 2005, 2010, or 2015. Eq 4.), a fitness ($F_{p_{i}}^{\left(n\right)}$) of a product *i* means the ratio between the number (*h*_*p*_) of competitive products and the dependency value and the average value of the fitness at the *n*-times of iterated calculation (Eqs 7 and 9). In other words, the product fitness $F_{p}^{\left(n\right)}$ could be the sum of countries inversely weighted with the dependency $D_{c}^{\left(n\right)}$, and this dependency would be the sum of products weighted with the fitness (Eqs 7–10).

This research establishes ${\tilde{F}}_{p}^{\left(0\right)}=1\;\forall p$ as the initial value of ${\tilde{F}}_{p}^{\left(0\right)}$. At the final step of the repeated calculation, this product fitness (Eqs 7 and 9) would reach a certain fixed point at *n* times of iterated calculation which is dependent upon the value about the dependency of trading partners at the *n*-1 times of the iteration. This research also sets ${\tilde{D}}_{c}^{\left(0\right)}=1\;\forall c$ as the initial value of ${\tilde{D}}_{c}^{\left(0\right)}$. The dependency of the partners (Eqs 8 and 10) implies that Korea has the revealed comparative advantage (RCA) of the exporting product with its importers as their dependent position of the product.

F˜pn=∑cQpcDcn−1 (7)

$${\tilde{D}}_{c}^{\left(n\right)}=\sum_{p}Q_{pc}F_{p}^{\left(n-1\right)}$$ (8)

※ Note. The initial conditions are ${\tilde{F}}_{p}^{\left(0\right)}=1\;\forall p$ and ${\tilde{D}}_{c}^{\left(0\right)}=1\;\forall c$.

Fpn=F˜pn<F˜pn> (9)

Dcn=D˜cn<D˜cn> (10)

※ Note. < · >: an average value of a metric in a pair of angle brackets.

**Fig 3:**
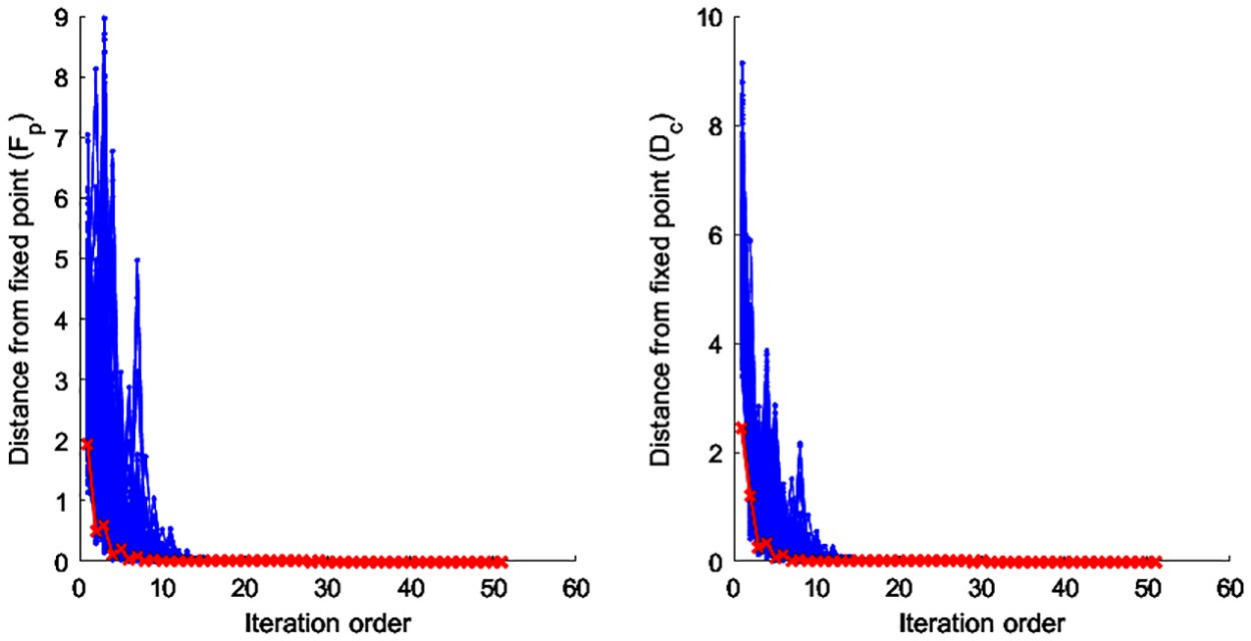
Evolution of *F*_*p*_ and *D*_*c*_ values at each iteration and distance with the iteration process of converged fixed points. ※ Notes. The red color shows the evolution paths of the initial values given in ${\tilde{F}}_{p}^{\left(0\right)}=1\;\forall p$ and ${\tilde{D}}_{c}^{\left(0\right)}=1\;\forall c$. This research reached a kind of fixed point with the conditions about fiftieth iteration and ***Q***_***pc***_ with p = 10, c = 27, ***p***_***h***_ = 0.6, and ***p***_***l***_ = 0.05.

**Fig 4:**
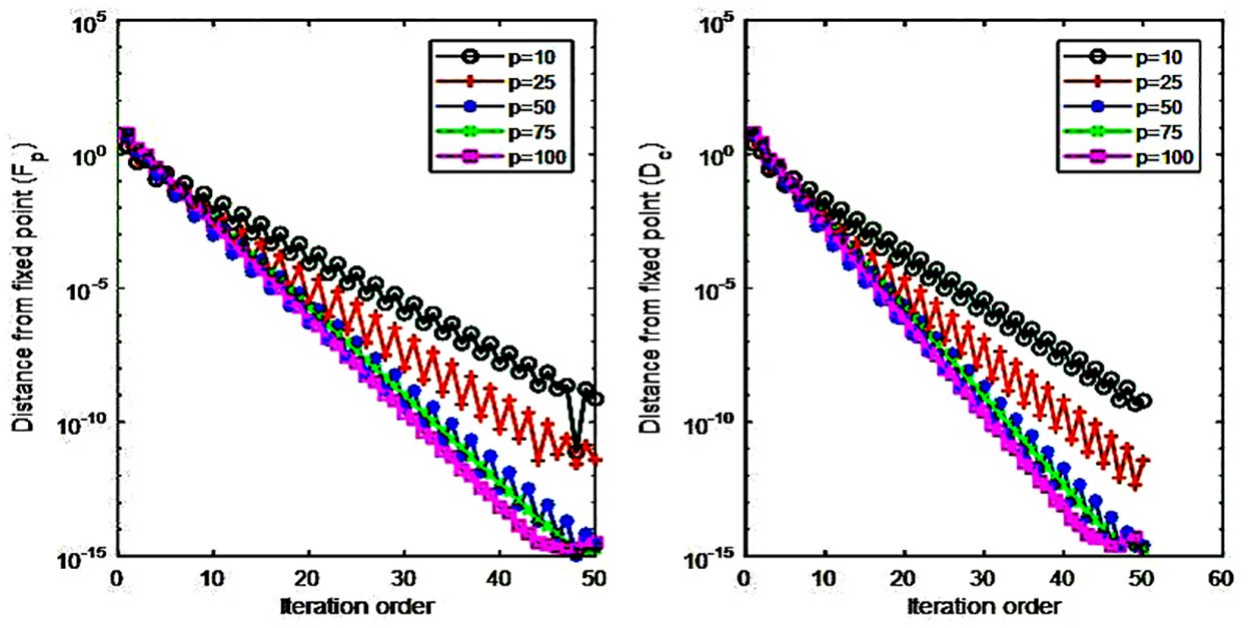
Exponential convergence on fixed points.

In summary, our nonlinear iteration method (NIM) for the calculation of the product fitness and the partner’s dependency consists of the following two steps. The first step calculates intermediate variables ${\tilde{F}}_{p}^{\left(n\right)}$ and ${\tilde{D}}_{c}^{\left(n\right)}$ (Eqs 7 and 8). The initial value of ${\tilde{F}}_{p}^{\left(0\right)}$ is one for all values of *p* and ${\tilde{D}}_{c}^{\left(0\right)}$ is one for all values of *c* (Eq 8). However, if dependency *D*_*c*_(*k*) of a partner *k* is zero, this research sets *D*_*c*_(*k*) as the multiplier of a value of *ρ* and the positive minimum value of *D*_*c*_ which the research selects the value among those of countries at the year (Eq 11). This research establishes the range of ρ as [0, 1] which could guarantee that *D*_*c*_(*k*) is the lowest value among all countries’ *D*_*c*_. Therefore, Eq 11 prevents the value of ${\tilde{F}}_{p}$ from its divergence because of the zero value of *D*_*c*_(*k*) and this impact on the iterative calculation of Eqs 7 and 8. The research sets the value of ρ as 0.9 and the corresponding results converge to a certain value (Figs 3 and 4). In the next step, the product’s fitness ${\tilde{F}}_{p}^{\left(n\right)}$ and a trading partner’s dependency ${\tilde{D}}_{c}^{\left(n\right)}$ could be calculated by normalizing ${\tilde{F}}_{p}^{\left(n\right)}$ and ${\tilde{D}}_{c}^{\left(n\right)}$.

$$D_{c}\left(i\right)=\rho \cdot min\left(D_{c}^{+}\right)$$ (11)

※ Notes. 0 < *ρ* < 1. This research sets the value of *ρ* as 0.9, which could support the convergence (i.e., fixed point) of the *F*_*p*_ and *D*_*c*_ (Please see the Result section).

### Sample data

This study obtained the paid data of exporting and importing Korean products for each trading partner from the website of Trade Statistics Services (TSS) which Korea Trade Statistics Promotion Institutes (KTSPI) manages [21] (Please see the Acknowledgment). This research categorized the products into 2- and 4-digit codes according to the Harmonized System 2007 (HS Code 2007) coding system [22]. This research employed a non-financial (or non-monetary) unit of data (e.g., the trade amount of products) in order for the realistic measurement for a nation’s production capabilities [8, 10]. The number of trading partners including countries and their territories varies each year but is around 200 in all cases (Table 1). This concrete identification of trading partners would be more accurate than nations because the identification can reflect the geological and continental location of the trading destination. The research analyzed datasets in the period of every five years (i.e., 1995, 2000, 2005, 2010, and 2015). Our final dataset for the analysis of *Q*_*pc*_ is a matrix with 97 products and 261 trading partners in accordance with 2-digit codes of HS Code 2007.

**Table 1:** The number of products and trading partners by year.

Row/Column of *Q*_*pc*_			1995	2000	2005	2010	2015	Total sum
Product	4-digit	Export	1,175	1,182	1,198	1,178	1,194	1,261 (rows)
		Import	1,222	1,229	1,233	1,215	1,219	
	2-digit	Export	97	97	97	97	97	97 (rows)
		Import	97	97	97	97	97	
Trading partners		Export	220	238	230	233	235	261 (columns)
		Import	206	226	225	232	243	

The aggregation of importing and exporting trading partners for each year did not exactly match the raw data due to the dynamics of import and export over time. If there was a pair of product-trading partner in a year, the pair were included in the analysis in order to generate the matrix of products and trading partners with the same size of the matrix during the period. As the result, this research finally identified 1,261 products with 4-digit codes (or 97 products with 2-digit codes) and 261 trading (i.e., importing and exporting) partners (Table 1). The import of 4-digit products was higher than the export in all years, and the number of importing trading partners gradually increased and eventually surpassed the number of exporting partners in 2015.

## Results

### Characteristics of the metrics’ fixed point

This study applies the iterative algebraic method that represents the coupled maps between *F*_*p*_ and *D*_*c*_ in order to measure the product’s fitness and the dependency between trading partners (Eqs 7–10). Before analyzing the characteristics of a measured value, it would be necessary to review the robustness of the NIM methodology. In other words, for the non-trivial fixed point to be valid for the given product-country matrix, the point must exist and be unique regardless of the initial value at the same time [12]. This research implemented a numerical simulation analysis similar to Cristelli, Gabrielli [12]’s methdology in order to confirm the fulfillment of the criteria. This research generates a random matrix *Q*_*pc*_ (Eqs 1–3 and Fig 2) and sets an initial value for the application of nonlinear iterative method (NIM), which is the revised version of Cristelli, Gabrielli [12]’ s methodology. In the case of a random matrix with the condition that *r* is the size of the matrix and *r* = *c*/*p* (i.e., *c* and *p* refer to the number of trading countries and products, respectively), the research sets *Q*_*ij*_ = 1 with the probability of *p*_*h*_ ≠ 0 if the elements of the ith row are *j* ≤ *ri*. Then, this study sets *Q*_*ij*_ = 1 if *p*_*l*_ < *p*_*h*_, *p*_*l*_ ≠ 0 and *j* > *ri*. In the case of the initial value (i.e., the matrix of *p* dimensions), the *p* − 1 number of points having uniform distribution at the interval [0, 1] is distributed, and the lengths of the resulting *p* sub-intervals are divided by their average lengths [23].

**Fig 5:**
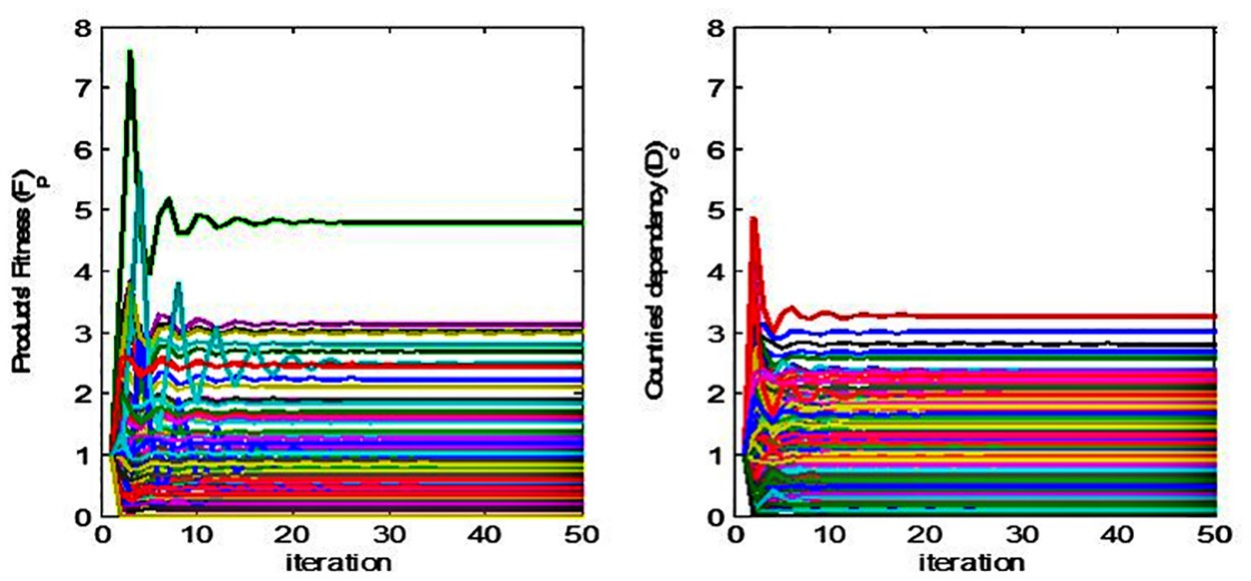
Evolution of the distribution of product’s fitness and trading partners’ dependency for the year of 2015.

This study sets *p*_*h*_ = 0.6, *p*_*l*_ = 0.05 for the triangular-shaped matrix *Q*_*pc*_ [12]. The study sets *r* = 2.7, which is similar to our final dataset of the matrix with *p* = 97 and *c* = 261 (i.e., *r* = 2.6907). The research gathered the sample of 100 matrices with the changes in *p* values of 10, 25, 50, 75, and 100. And then, the research analyzed 1,000 random initial values of each matrix. The following results (Figs 3 and 4) show that all cases of the initial values with the matrix size *r* converge on a certain level of their fixed points. Fig 5 shows the evolution of coupled maps between the product’s fitness and the dependency of trading partners generated from the matrix in 2015. The other periods also show a qualitatively similar evolution pattern.

### Changes in product fitness and country’s dependency

#### Variation of product fitness

This section explains the consequences of product fitness and trading partners’ dependency in order of descriptive statistics (i.e., annual correlation), top ten members of products and partners, and several selected products and partners. Above all, this research analyzed the Pearson correlation for small or medium-sized sample values in order to investigate statistically the product’s fitness change by year. Since the correlation between the product fitness (i.e., fit95, fit00, …, fit15) of 96 products is at least 76.7% (Table 2), the interrelationship of the product’s fitness value in each year is considerably linear. Therefore, the structural change of the fitness of Korean products every five years seems to be somewhat smaller than the time change of every five years in the linear distribution of total volumes. The ratio of the amount of export to that of import (i.e., exim95, exim00, …, exim15. Eq 12.) of Korean products has correlation of at least 65.6% in each year (Table 2). However, the correlation to the product’s fitness is a maximum of 33.1% (exim00-fit10 in Table 2) in the same period, indicating a relatively low linear relationship. The results of Kendall’s Tau correlation are also very similar to the results of Pearson’s correlation.

**Table 2:** Pearson correlation between “product’s fitness” and “ratio of export to import”.

	**fit95**	**fit00**	**fit05**	**fit10**	**fit15**	**exim95**	**exim00**	**exim05**	**exim10**	**exim15**
**fit95**										
**fit00**	0.92									
**fit05**	0.87	0.92								
**fit10**	0.81	0.86	0.92							
**fit15**	0.77	0.83	0.88	0.91						
**exim95**	0.20	0.26	0.23	0.15	0.19					
**exim00**	0.26	0.32	0.33	0.34	0.31	0.66				
**exim05**	0.23	0.32	0.31	0.26	0.29	0.82	0.81			
**exim10**	0.19	0.28	0.28	0.21	0.25	0.78	0.76	0.99		
**exim15**	0.21	0.32	0.32	0.27	0.28	0.69	0.83	0.96	0.97	

※ Note. All numbers are rounded to three decimal places.

The product’s fitness could have different information characteristics than the relative portion of export. Furthermore, the ranking of the top ten percentage (or 10 products) in product’s annual fitness indicates that vehicles other than railway or tramway rolling-stock [product id (hereinafter id) 87] and parts and accessories thereof and Rubber and articles thereof (id 40) are leading competitive in all years (Table 3). However, that the fitness of these two products tends to decrease gradually.

$$exim_{it}=\left(Q_{it}^{export}\right)/\left(Q_{it}^{import}\right)$$ (12)

※ Notes. *i*: product id. *t*: the year of 1995, 2000, 2005, 2010, or 2015.

**Table 3:** List of products in the top 10 products in fitness.

**Ranking**	**1995**		**2000**		**2005**	
	**(Product id) Products**	**Fit**.	**(Product id) Products**	**Fit**.	**(Product id) Products**	**Fit**.
1	(87) Vehicles	6.42	(87) Vehicles	5.45	(87) Vehicles	5.12
2	(40) Rubber	5.79	(40) Rubber	4.93	(40) Rubber	4.45
3	(55) Man-made staple	3.15	(54) Man-made filaments	2.51	(39) Plastic	3.39
4	(84) Nuclear reactors	2.96	(39) Plastic	2.50	(30) Pharmaceutical	2.89
5	(90) Optical	2.73	(90) Optical	2.42	(49) Printed books	2.88
6	(96) Miscellaneous manufactured	2.48	(30) Pharmaceutical	2.31	(72) Iron and steel	2.40
7	(49) Printed books	2.41	(89) Ships	2.31	(89) Ships	2.38
8	(54) Man-made filaments	2.38	(55) Man-made staple	2.17	(54) Man-made filaments	2.29
9	(56) Wadding	2.3	(84) Nuclear reactors	2.14	(84) Nuclear reactors	2.25
10	(39) Plastic	2.15	(49) Printed books	2.10	(85) Electric machinery	2.19
Ave.	-	1.01	-	1.01	-	1.01
Med.	-	0.78	-	0.73	-	0.68
Var.	-	1.06	-	0.85	-	0.83
**Ranking**	**2010**		**2015**		**5-year’s Average**	
	**(Product id) Products**	**Fit**.	**(Product id) Products**	**Fit**.	**(Product id) Products**	**Fit**.
1	(87) Vehicles	4.86	(87) Vehicles	4.82	(87) Vehicles	5.33
2	(40) Rubber	3.90	(40) Rubber	3.15	(40) Rubber	4.45
3	(30) Pharmaceutical	3.57	(49) Printed books	3.04	(39) Plastic	2.84
4	(39) Plastic	3.32	(84) Nuclear reactors	3.01	(30) Pharmaceutical	2.70
5	(89) Ships	3.30	(39) Plastic	2.83	(84) Nuclear reactors	2.69
6	(84) Nuclear reactors	3.10	(30) Pharmaceutical	2.70	(49) Printed books	2.61
7	(49) Printed books	2.62	(89) Ships	2.51	(89) Ships	2.48
8	(95) Toys, games	2.26	(38) Miscellaneous chemical	2.50	(54) Man-made filaments	2.15
9	(94) Furniture	2.16	(95) Toys, games	2.48	(55) Man-made staple	2.08
10	(73) Articles of iron/ steel	2.12	(22) Vegetables, fruits	2.26	(95) Toys, games	1.97
Ave.	–	1.01	–	1.01	–	1.01
Med.	–	0.70	–	0.70	–	0.81
Var.	–	0.85	–	0.72	–	0.76

※ Notes. All numbers are rounded to three decimal places. This research removed product (id 99) because of its trivial value of 0. Fit: Fitness. Ave: Average. Med.: Median. Var: Variance.–: not available.

#### Differences in trading partners’ dependency

The dependency of 261 trading partners on Korea had a linear relationship of a minimum of 62.3% (Table 4). The linear correlation of relative export portion (minimum 87.1%) was high except for 2000, which was right after the Asian financial crises during the period 1997–1998. The mutual linear distribution between the two variables was a maximum of -8.9%, indicating a very low linear relationship. The results of Kendall’s Tau rank correlation are also very similar to the results of Pearson’s correlation.

**Table 4:** Pearson correlation between “dependency of trading partner country” and “ratio of export to import”.

	**dep95**	**dep00**	**dep05**	**dep10**	**dep15**	**exim95**	**Exim00**	**exim05**	**exim10**	**exim15**
**dep95**										
**dep00**	0.81									
**dep05**	0.77	0.81								
**dep10**	0.70	0.71	0.79							
**dep15**	0.63	0.63	0.72	0.81						
**exim95**	-0.05	-0.02	-0.03	-0.02	-0.06					
**exim00**	-0.04	-0.10	-0.09	-0.08	-0.09	0.01				
**exim05**	-0.08	-0.03	-0.08	-0.07	-0.08	0.88	0.00			
**exim10**	-0.09	-0.04	-0.08	-0.09	-0.09	0.88	0.08	1		
**exim15**	-0.09	-0.04	-0.09	-0.08	-0.08	0.88	0.01	1	0.99	

※ Note. All numbers are rounded to three decimal places.

**Fig 6:**
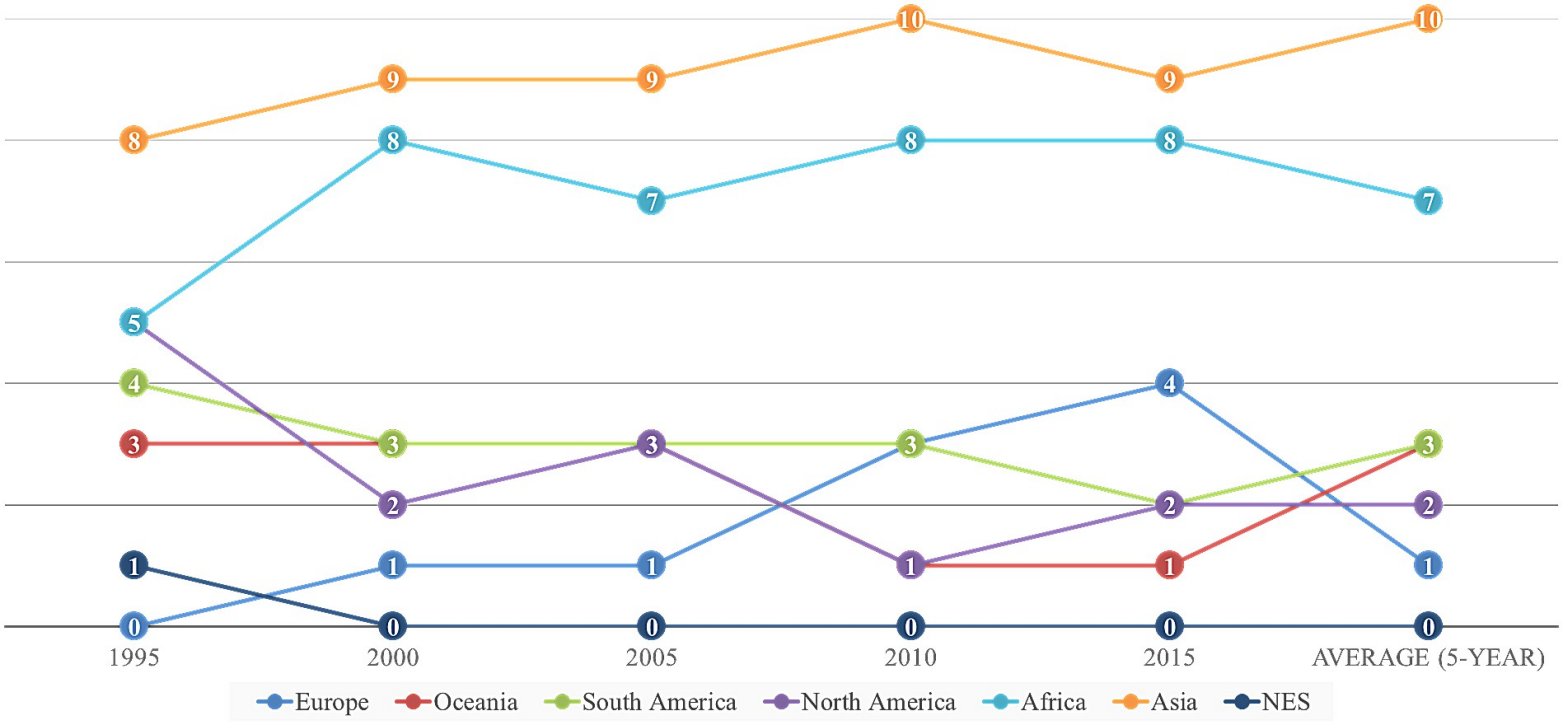
Regional distribution of the dependency of the top 10% (or 26) trading partners. ※ Note. NES: Not Elsewhere Specified.

The physical distance from Korea to the region can affect a trading partner’s dependence on Korea. This feature can raise the need to examine the frequency of the region of countries in the top ten partners in terms of dependency. Asia and Africa show the highest dependency among the regions (Fig 6). The regional distribution of 5-year average is generally similar in order of Asia (i.e., 10 trading partners), Africa (seven partners), South America (three), Oceania (three), North America (two), and Europe (one) (Table 5).

**Table 5:** List of countries in the top 10 trading partners in terms of dependency with the years of 1995, 2000, 2005, 2010, and 2015.

**Rk**	**1995**			**2000**			**2005**		
	**Nation**	**Dep**	**Reg**	**Nation**	**Dep**	**Reg**	**Nation**	**Dep**	**Reg**
1	Mongolia	3.060	ASA	Fiji	2.576	OCE	Pakistan	2.614	ASA
2	New Zealand	2.713	OCE	Russian Fed.	2.572	EUR	Lebanon	2.610	ASA
3	Argentina	2.608	SAM	Pakistan	2.530	ASA	Argentina	2.534	SAM
4	Bahrain	2.579	ASA	Senegal	2.439	AFA	Indonesia	2.483	ASA
5	Australia	2.575	OCE	Mongolia	2.433	ASA	Dominican Rep.	2.472	NAM
6	Viet Nam	2.461	ASA	Yemen	2.378	ASA	Senegal	2.469	AFA
7	Libya	2.287	AFA	New Zealand	2.363	OCE	New Zealand	2.442	OCE
8	Yemen	2.260	ASA	Egypt	2.346	AFA	Libya	2.433	AFA
9	Indonesia	2.236	ASA	Canada	2.261	NAM	Mauritius	2.395	AFA
10	Rep. of South Africa	2.232	AFA	Rep. of South Africa	2.254	AFA	Peru	2.358	SAM
	Average	1.000	–	Average	1.000	–	Average	1.000	–
	Median	1.000	–	Median	1.004	–	Median	1.017	–
	Variance	0.517	–	Variance	0.483	–	Variance	0.538	–
**Rk**	**2010**			**2015**			**5-year’s Average**		
	**Nation**	**Dep**	**Reg**	**Nation**	**Dep**	**Reg**	**Nation**	**Dep**	**Reg**
1	Mongolia	2.742	ASA	Mongolia	3.280	ASA	Mongolia	2.708	ASA
2	Russian Fed.	2.473	EUR	Russian Fed.	3.007	EUR	Pakistan	2.351	ASA
3	Lithuania	2.455	EUR	Kenya	2.827	AFA	New Zealand	2.312	OCE
4	Fiji	2.446	OCE	Kazakhstan	2.680	ASA	Russian Fed.	2.245	EUR
5	United Arab Emirate	2.374	ASA	Guam	2.625	NAM	Argentina	2.201	SAM
6	Guatemala	2.326	NAM	Myanmar	2.593	ASA	Bolivia	2.154	SAM
7	Sri Lanka	2.300	ASA	Kyrgyzstan	2.411	ASA	Kenya	2.124	AFA
8	Guinea	2.294	AFA	Tanzania	2.395	AFA	Rep. of South Africa	2.104	AFA
9	Pakistan	2.283	ASA	Pakistan	2.392	ASA	Tanzania	2.065	AFA
10	Kazakhstan	2.225	ASA	Ukraine	2.368	EUR	Peru	2.018	SAM
	Average	1.000	–	Average	1.000	–	Average	1.000	–
	Median	1.009	–	Median	0.951	–	Median	1.007	–
	Variance	0.516	–	Variance	0.552	–	Variance	0.409	–

※ Notes. Rk: Ranking. Dep: Dependency. Reg: Region.–: not available. AFA: Africa. ASA: Asia. EUR: Europe. NAM: North America. OCE: Oceania. SAM: South America.

### Industries’ competitiveness and regional dependency

It would be necessary to match the products with the most suitable industry in order to analyze them at the industry level. This research collected the import and export data in accordance with the product categorization standard of HS Code 2007. The research allocated each product to the most suitable industry using the International Standard Industrial Classification (ISIC) on all economic activities which United Nations Statistical Division (UNSD) offers; it rearranged the products into 14 industries as shown in Table 6 (i.e., I-01, I-02, …, I-14). This research integrated trading partners into 6 regions: Africa (R-1), North America (R-2), South America (R-3), Asia (R-4), Europe (R-5), and Oceania (R-6). As shown in Eqs 13 and 14, the industry’s fitness *F*_*s*_ is the weighted average of fitness *F*_*p*_ of the matched products (*p*), whereas dependency *D*_*r*_ of the global region is the weighted average of dependency *D*_*c*_ of trading partners (*c*) [3].


Fs=∑p∈SwpFp∑p∈Swp (13)

Dr=∑c∈RwcDc∑c∈Rwc (14)

**Table 6:** Classification of fourteen industries based on ISIC.

Fourteen industries based on ISIC	96 products based on HS Code 2007
(I-01) Agriculture/Forestry, fishing and mining	01, 05, 06, 07, 08, 09, 10, 12, 13, 14, 25, 26
(I-02) Manufacture of food products/beverages and tobacco products	02, 03, 04, 11, 15, 16, 17, 18, 19, 20, 21, 22, 23, 24
(I-03) Manufacture of textiles and leather products	41, 42, 43, 50, 51, 52, 53, 54, 55, 56, 57, 58, 59, 60, 61, 62, 63, 64, 65
(I-04) Manufacture of wood/paper products, printing and reproduction of recorded media	44, 45, 46, 47, 48, 49
(I-05) Manufacture of coke and refined petroleum products	27
(I-06) Manufacture of chemicals, chemical and rubber/plastic products	28, 29, 30, 31, 32, 33, 34, 35, 36, 37, 38, 39, 40
(I-07) Manufacture of non-metallic mineral products	68, 69, 70
(I-08) Manufacture of basic metals	72, 74, 75, 76, 78, 79, 80, 81
(I-09) Manufacture of fabricated metal products	73, 82, 83
(I-10) Manufacture of machinery and equipment (n.e.c.)	84, 93
(I-11) Manufacture of electrical and electronic equipment	85
(I-12) Manufacture of precision instruments	90, 91
(I-13) Manufacture of transport equipment	86, 87, 88, 89
(I-14) Manufacture of other manufacturing (n.e.c.)	66, 67, 71, 92, 94, 95, 96

※ Note. n.e.c.: not elsewhere classified.

Weighted averages *w*_*p*_ and *w*_*c*_ were assumed to be proportional to the export volume ($Q_{p}^{export}$ and $Q_{c}^{export}$, respectively) by reflecting the fact that fitness and dependency are related to the import and export volumes and diversification (Eqs 15 and 16). Parameter *σ* incorporates the adverse effect of import and represents the ratio of export to the total trade volume (i.e., the sum of trade volumes about export and import).


$$w_{p}=\sigma Q_{p}^{export}=\frac{Q_{p}^{export}}{\left(Q_{p}^{export}+Q_{p}^{import}\right)}Q_{p}^{export}$$ (15)

$$w_{c}=\sigma Q_{c}^{export}=\frac{Q_{c}^{export}}{\left(Q_{c}^{export}+Q_{c}^{import}\right)}Q_{c}^{export}$$ (16)

**Fig 7:**
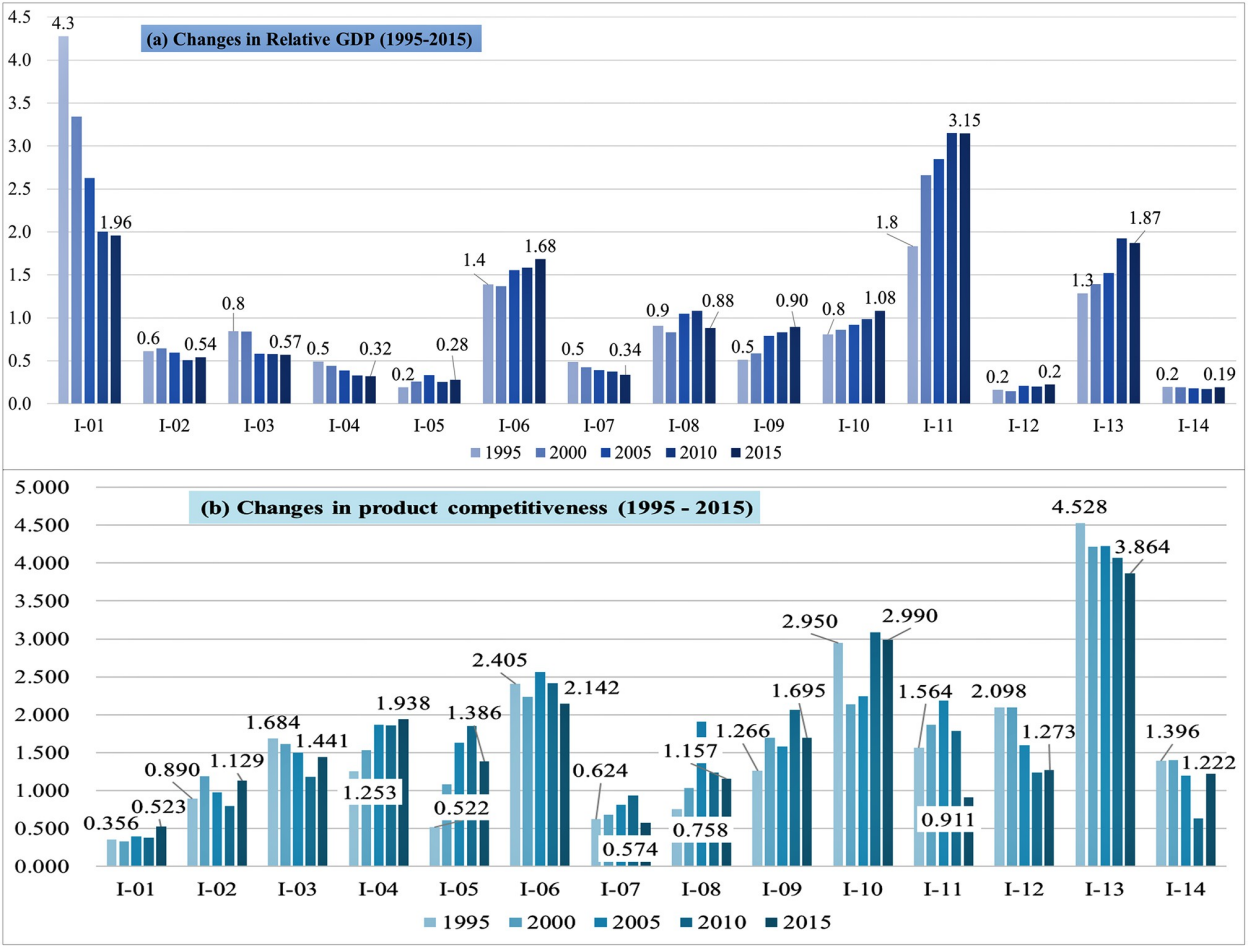
Evolution of the industry’s relative GDP and competitiveness. ※ Note. Please see Table 6 for the classification of the 14 industries (e.g., I-01, I-02).

Fig 7 shows the evolution of the industry’s relative GDP and competitiveness evaluated with the methodology described above. This research measures the relative value of GDP as the value normalized with total average in order to have the relative characteristics in the same way as in the competitiveness comparison.

In GDP, I-01 (agriculture/forestry, fishing, and mining), I-06 (chemical), I-11 (electrical and electronic equipment), and I-13 (transport equipment) industries belonged to top one group throughout the analysis period (1995–2015). These groups accounted for more than 60% of the total production. These notable changes denote the finding that the I-11 industry surpassed I-01 to take first place from 2004, and the I-13 industry increased steadily to raise its ranking in third place. Although the top two groups included the I-03 (textile), I-08 (basic metals), and I-10 (machinery and equipment) industries at the beginning of the analysis period, the I-09 (fabricated metal products) industry has replaced I-03.

Our relational evaluation shows different results among industry sectors. The I-11 industry, which ranked in first place with its production volume in the latter part of the analysis, showed a pattern of declining competitiveness during the same period, implying the instability of GDP growth as a monetary index. The I-13 industry showed a relatively high level of competitiveness from the diversification viewpoint throughout the analysis period. However, the declining pattern of competitiveness and the production stagnation of during the analytic period imply the need for improving industrial and export competitiveness by increasing the trade volume or the market diversification. I-01 and I-12 (precision instruments) have shown the opposite evolution patterns. I-01 had high but rapidly decreasing production size, whereas relational competitiveness has steadily increased. I-12 had gradually increased production size, but relational competitiveness has steadily decreased.

**Fig 8:**
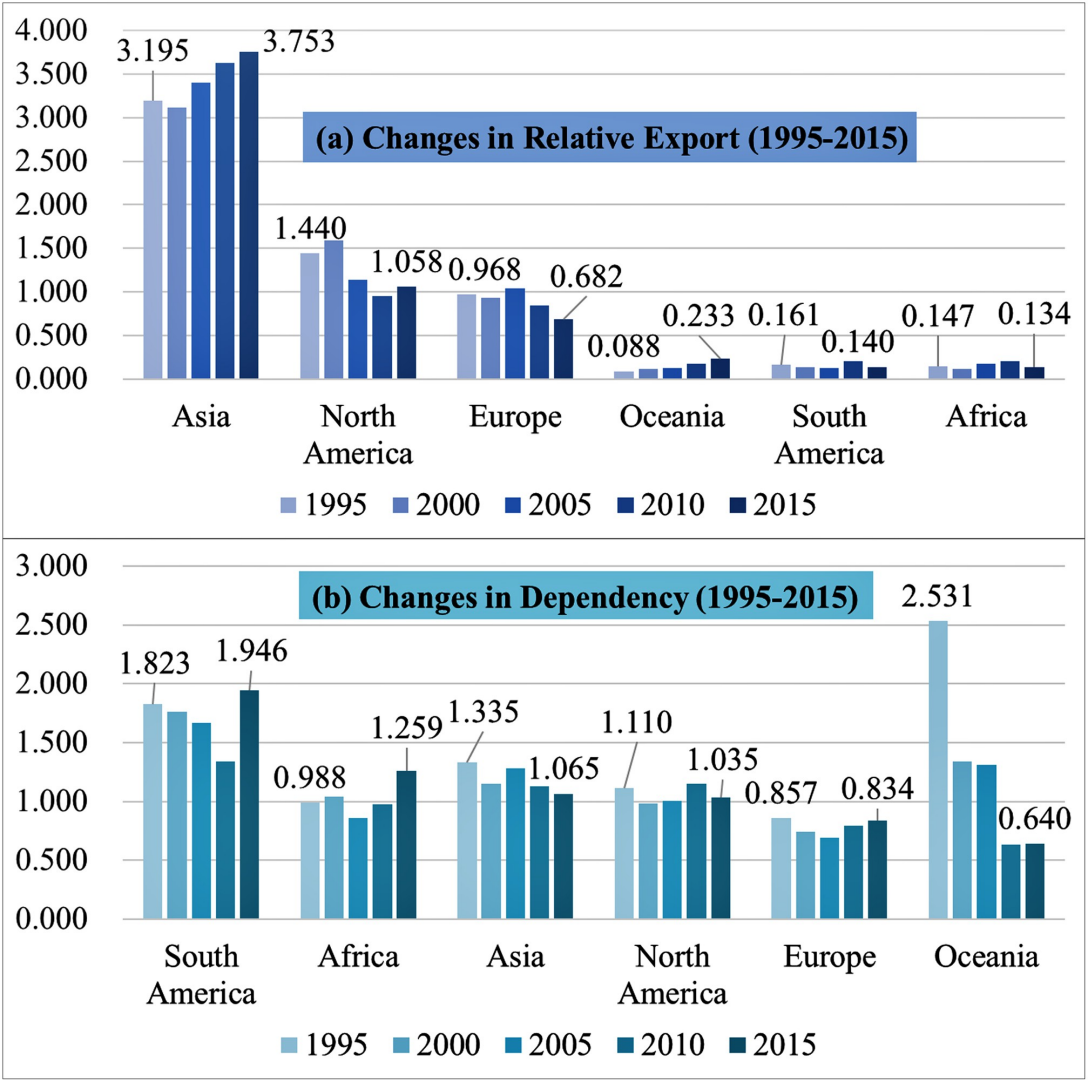
Evolution of the region’s relative export and dependency.

The evaluation of dependency of trading regions shows a notable decline in the Oceania (R-6) region (Fig 8). Although the export volume to this region is not large, it is gradually increasing. Its decreasing dependency on Korea indicates that the items exported to the region appear to be relatively uncompetitive and their proportion seems to be large. The Asian region (R-4), which accounts for more than half of Korea’s total trade volume, shows a similar pattern. While the total export volume (i.e., 50–60% of the sum value) is rapidly increasing, the region’s dependency on Korea seems to be somewhat diminishing.

## Discussion

This study utilized the import-export data of products and trading partners and analyzed the competitiveness of Korean products and dependency of partners in the global market with respect to two research questions. As the answer to **RQ1** with regard to “product competitiveness”, Korea’s top ten export-competitive products were vehicles (id 87), rubbers (id 40), fibres (id 55), nuclear reactors (id 84), optical and relevant parts (id 90), manufactured articles (id 96), printed materials (id 49), filament (id 54), wadding articles (id 56), and plastic articles (id 39) in Table 3. They showed relatively small change during the period 1995–2015.

As the answer to **RQ2** with regard to the analysis of major trading partners, those with high “dependency” on Korea were mostly in Asia—which was physically close to Korea—and Africa as developing countries. These results imply that Korea’s trading pattern had the status quo aspect of concentrating on specific products and partners.

The existing global analysis evaluates the competitiveness of products exported by a country based on the relative volume [8, 9]. This study’s local analysis considers not only the evaluation of the relative volume of each product but also the diversification of trading countries. It would be important for a country to secure robustness amid external impact through diversification under the dynamic environment of international competition and technology innovation. This diversification strategy with dynamics can become one of the important explanatory factors describing national or industrial competitiveness and wealth [8].

Moreover, the findings of this research show some differences of trade dependencies in regions and transactional partners. The top 10% trading partners were in the regions of Asia and South America (Fig 6). On the other side, the regional dependence by Korea’s trade was high for South America, Africa, and Asia (Fig 8). Some geographical and geopolitical factors may affect this discrepancy of regional dependence between partners and regions. For example, some Asian nations (e.g., Mongolia, Pakistan, and Indonesia in Table 5) have high tendency of geographical proximity (e.g., the Strait of Malacca as the choke position of international trades in Asia [24]) and similar background in oriental cultures with Korea. However, the proportion of top 10% trading partners in Asia is comparatively low, which could be the results of socioeconomic competition between influential countries (e.g., China as Group two (G2), Japan) and the increasing trading portion of other regions (e.g., South America, Africa) with economic growth.

Fig 8 also denotes that the Asian region has a high portion of trade to Korea but a low level of dependence in comparison with other regions, which could suggest the unique features of the Asian region. The Asian region has achieved rapid economic growth and political stabilization; thus, it has consequently realized its higher level of trade diversification than the other developing countries [25]. In addition, the hegemonic competition/conflicts between global influential nations (e.g., USA, China, Russia, and Japan) has been accelerated in the Northeastern Asia, referred to the “Cold War” and “New Cold War [26, 27].” However, many developing countries in South America and Africa has slowly achieved their economic advances with their (inter)national conflicts of politics and war. Their trade volume might be smaller than those in Asia and their transactional dependence on Korea could be higher than those in Asia.

Therefore, this study suggests the need to diversify the trade policies and industrial development strategies at the national level whenever the current comparative advantage of product export and distribution of major trading partners are maintained or strengthened. For example, the Asian financial crisis (1997–1998) and the US-initiated economic crisis (2007–2008) had been influenced on the world trade as these events had driven nations to changes of their dependence to specific regions and products.

Our results of the competitiveness of Korean products and its dependency about trading countries focusing on particular products and regions (during times of crises) will raise concerns about the long-term technological and industrial adaptability and sustainability of trade strategy of Korea. In this turbulent era of the Fourth Industrial Revolution and the global economic shocks of pandemics (e.g., SARS in 2003, MERS in 2012, and COVID-19), Korea, and other similar export-oriented nations need to diversify the transactional products and trading partners for their dynamic changes and future in the medium and long-term future.

## Conclusion

It could be very important for a country to diversify and upgrade its industrial structure and trade products for the country to attain stable long-term economic development in its global competition. This study configured the matrix of products and trading partners from Korea’s trade data of each product and trading country during the period 1995–2015. The study analyzed the product’s competitiveness and trading partners’ dependence using revealed comparative advantage (RCA) and nonlinear iterative method (NIM). These results showed that Korea had continuously maintained its competitiveness with specific products (e.g., electrical and electronic equipment, chemicals, and transport equipment) even during the Asian and US-initiated financial crises; many developing countries in Asia, Africa, and South America were major trading partners. This research suggests the necessity of the strategy of diversification of traded products, trading countries, and regions as one of the industrial and trade policies for Korea and export-oriented countries with their narrow domestic market or insufficient natural resources.

This study implements an analytical approach based on the data from a specific country, which would be a limiting point in the generalization of results in comparison with the competitive trade and industrial strategies of other countries. It would be necessary and important to analyze the competitiveness of the traded products and the complexity of trade relationship within the complex trade relationship of the network. Moreover, sustainable economic development with the problems of global warming and environmental destruction has already become a global issue as an international concern [28, 29]. The analysis of national and regional industrial structure and the ripple effect from the trade relationships between the supply and demand of inter-industry sectors would be also important, as some of the subjects in future research. For example, energy and natural resources, which would not be replaceable in the short-term between countries in geographical proximities, could be strategic trading goods for nations in the era of the “New Cold War” provoked by military conflicts in Ukraine in 2022 and the consequent inflation of imported goods due to the recovery from COVID-19 and highly inter-dependent networks of international trade.
