# Correction: Global trade of South Korea in competitive products and their impact on regional dependence

**DOI:** 10.1371/journal.pone.0318434

**Published:** 2025-01-24

**Authors:** The PLOS ONE Staff

The first two authors, Dongsuk Kang and Pil-sun Heo should be noted as shared first co-authors on this work instead of all authors.

The publisher apologizes for the errors.
